# Nasopharyngeal microbiota evaluation in three cohorts of children in the Romanian pediatric population

**DOI:** 10.1186/1471-2334-14-S7-O21

**Published:** 2014-10-15

**Authors:** Anca Streinu-Cercel, Oana Săndulescu, Ioana Berciu, Alina Cristina Neguț, Mihai Săndulescu, Ruxandra Aursulesei, Adrian Streinu-Cercel

**Affiliations:** 1 Carol Davila University of Medicine and Pharmacy, Bucharest, Romania; 2 National Institute for Infectious Diseases "Prof. Dr. Matei Balş", Bucharest, Romania; 3 Dental Concept Studio, Bucharest, Romania

## Background

The nasopharyngeal microbiota represents one of the key factors related to infectious diseases in children [1]. The infectious agents and their resistance patterns are main factors driving disease severity. Nasopharyngeal carriage is high in children, especially for *Staphylococcus aureus*[2,3].

## Methods

We performed a screening study for nasopharyngeal carriage of *Staphylococcus* spp. in immunocompetent children aged 7-10 years old, attending a community school in central Bucharest (group 1), and in two groups of immunosuppressed children: children with hemato-oncologic diseases (lymphoma/leukemia) admitted to the Fundeni Clinical Institute, Bucharest (ages 2-10 years, group 2), and institutionalized children with vertically transmitted HIV infection, from the National Institute for Infectious Diseases “Prof. Dr. Matei Balş”, Bucharest (ages 1-10 years, group 3).

## Results

We analyzed data from 139 pharyngeal swabs (35.3% in group 1, 56.1% group 2 and 8.6% group 3), and 143 nasal swabs (37.1% group 1, 54.5% group 2 and 8.4% group 3). Pharyngeal cultures were positive for *Staphylococcus* spp. in 28.6% of children in group 1, 11.5% in group 2 (p=0.00755 vs. group 1) and 16.7% in group 3 (p=0.20045 vs. group 1). Of the positive pharyngeal samples, 92.9% were *S. aureus* in group 1, 100% in group 2 and 100% in group 3.

Nasal cultures were positive for *Staphylococcus* spp. in 84.9% of children in group 1, 48.7% group 2 (p=0 vs. group 1) and 50.0% group 3 (p=0.00391 vs. group 1). Of the positive nasal samples, 62.2% were identified as *S. aureus* in group 1, 94.6% in group 2 (p=0.00027 vs. group 1) and 66.7% in group 3.

## Conclusion

Pharyngeal carriage of *Staphylococcus* strains was low, however when positive, most strains were *S. aureus*. Nasopharyngeal carriage was significantly higher in immunocompetent children from the community compared to immunodepressed children. When present, *S. aureus* had a higher prevalence compared to coagulase-negative staphylococci (CoNS), particularly in immunodepressed children.
